# Anxiety, Practice Modification, and Economic Impact Among Iraqi Dentists During the COVID-19 Outbreak

**DOI:** 10.3389/fmed.2020.595028

**Published:** 2020-12-21

**Authors:** Anas F. Mahdee, Sarhang S. Gul, Ali A. Abdulkareem, Syed Saad B. Qasim

**Affiliations:** ^1^Department of Restorative and Aesthetic Dentistry, College of Dentistry, University of Baghdad, Baghdad, Iraq; ^2^Department of Periodontics, College of Dentistry, University of Sulaimani, Sulaimani, Iraq; ^3^Department of Periodontics, College of Dentistry, University of Baghdad, Baghdad, Iraq; ^4^Department of Bioclinical Sciences, Faculty of Dentistry, Kuwait University, Safat, Kuwait

**Keywords:** anxiety, coronavirus, Iraqi dentists, economic, practice management

## Abstract

**Objectives:** As health care workers on the front line during the coronavirus (COVID-19) pandemic, dental practitioners are amongst those at risk due to their close contact with potentially infected individuals. The aim of the current study was to assess the anxiety, awareness practice modification, and economic impact amongst Iraqi dentists whilst working during the outbreak.

**Methods:** This study was performed using an online survey questionnaire with aid of Google forms from 2nd to 23rd July 2020. A total of 484 clinicians responded. The questionnaire was composed of open end, closed end, and Likert five-point scale questions to assess anxiety, awareness and financial impact of COVID-19 on dentists. Mann–Whitney test was used to compare two groups, whilst Kruskal–Wallis was performed by *post-hoc* test for multigroup comparisons.

**Results:** The mean age of participants was 36.51 ± 9.164 years and the majority (75.2%) of these were graduate dentists only. More than 80% of participants reported anxiety of catching COVID-19. The recorded anxiety level was higher amongst younger dentists and females. Awareness and practice levels among these dentists of precautions and infection-control measures associated with COVID-19 (94%) was found to be high and to be statistically significantly affected by age, qualification and designation (except GP vs. Specialist). With respect to the economic impact, about 75% of practitioners, regardless of demographical variables, reported that their income had declined by about 50%.

**Conclusions:** The investigation provides clear insights into the anxiety, practice modifications and economic impact on dentists working in Iraq. Although there is a high level of knowledge and awareness of required practice regarding the COVID-19 outbreak among Iraqi dentists, they also reported a high level of anxiety.

## Introduction

Since the emergence of the novel coronavirus disease (COVID-19) in Wuhan, China, all aspects of life have been influenced worldwide. The COVID-19 pandemic has spread in an exponential manner (1, 2), affecting millions of people worldwide and causing hundreds of thousands of deaths (3). Many countries have shut down their teaching institutes, industries, sport activities, social gatherings, public events, and airports. Drastic measures such as individual self-quarantine and social distancing rules have been introduced in an attempt to control the spread of the infection (4). The condition in Iraq has been no better than in many other countries. Since the first registered case of COVID-19 was recorded on the 24th of February 2020 in an Iranian student attending the city of Najaf in the south of Iraq, the spread of the virus has been escalating and as of 1st of July there were 51,524 confirmed cases and 2,050 deaths.

COVID-19 belongs to the Coronaviridae virus family, which is characterized by a single strand RNA structure (5). This virus has potential to cause severe respiratory tract infection and pneumonia among infected individuals, and can be easily transmitted via hand contact, saliva, nasal droplets and contaminated surfaces (2, 6). Health care workers and dentists in particular are categorized as at high risk of catching this infection (7). This could be because their close contact with patients during routine dental procedures increases the possibility of infection transmission (8, 9). Droplets and aerosols that are generated during dental procedures by such as high speed handpieces, air-water syringes, and ultrasonic scaling could produce a contaminated pathogenic environment within the dental working field during treatment of an infected person (6, 9, 10). Therefore, the risk of infection transmission within the dental team cannot be controlled through the standard protective measures of daily dental practice (8). This categorization as high-risk professionals could increase fear within the dental community (11, 12).

Regarding the rapid spread of the infection, the World Health Organization (WHO) and American Dental Association (ADA) published specific precaution guidelines to be implemented by dentists during treatment of urgent and emergency cases only. Otherwise, they stipulated that dental offices should be kept closed during the outbreak (13, 14). These guidelines emphasized the use of the appropriate precautions including wearing personal protective equipment (PPE) during dental procedures (13, 14). In addition, the use of antibacterial mouth washes, rubber dam, and high-volume section during treatment procedures with frequent cleaning and disinfecting of surfaces of chairs, door handles and floors was highly recommended (8, 15). Providing a secure environment is of paramount importance for dentists and dental staff to conduct dental work in a safe working environment. The high infection rate of COVID-19 and lack of PPE might affect the anxiety of the dentist. The levels anxiety of dentists have shown a negative impact on decision making, quality of work, and burnout (16).

On the other hand, in dental practice the demand arose for more expensive aerosol controlling equipment such as the high efficiency particulate arrestor (HEPA) during the outbreak period (10). The expense of these precautionary measures and limitations in the treatment of patients may have had serious economic impact on the dentistry field. Hence, the future careers of dentists could be affected if the outbreak continues for an indefinite period.

Meanwhile, no study has assessed the levels of anxiety among Iraqi dentists in the wake of the COVID-19 outbreak, their awareness about this illness and the infection control guidelines to prevent its spread, or the financial impact on their current practice. Therefore, the aim of this study was to use a specially designed online-based questionnaire to assess the impact of the COVID-19 outbreak on Iraqi dentists in terms of their anxiety, awareness and practice modification, and the financial implications for their dental practice.

## Methods

### Survey Administration

A cross-sectional survey was conducted using an online questionnaire that was electronically sent to Iraqi dentists by Iraqi Dental Association (IDA). Administration of the questionnaire started on 02/07/2020 for a period of 2 weeks, which was extended for another week after a reminder was sent, and ended on 23/07/2020. The study was approved by the ethics committee of the College of Dentistry, University of Baghdad in compliance with the Helsinki declaration (Ref No. 579/06/2020).

### Study Population and Sample Size

The survey was exclusively sent to registered Iraqi dentists and only completed forms were included in the final analysis. All incomplete forms and those returned outside the required timeframe were excluded.

The total number of registered Iraqi dentists, as officially provided by the IDA, was 6,463. Sample size was determined according to the following formulas (17, 18):

$$\begin{array}{ll} Sample\;size & =\left(distribution\;of\;50\%\right)/[(margin\;of\;error\%/\\ \; & \;confidence\;level\;score{)}^{2}]\\ Confidence\;level & =1.96\;\left(for\;confidence\;level\;of\;95\%\right),\\ \; & \;margin\;of\;error\;=\;0.05.\\ True\;sample & =\left(sample\;size\;\times \;population\right)/\\ \; & \;\left(sample\;size\;+\;population\;-\;1\right) \end{array}$$

The calculated sample size was equal to 363 which was further adjusted to take account of the dropout risk, previously estimated from a pilot study, by using the following formula:

$$\begin{array}{l} \text{N}=\text{n}/\left[1-\left(\text{z}/100\right)\right] \end{array}$$

Where *N* is the adjusted sample size, *n* is the calculated sample size, *z* represents the hypothesized attrition rate (25%). The final sample size was equal to 484 dentists. Accordingly, the questionnaire link was distributed via emails to corresponding number of randomly selected dentists.

### Questionnaire Design

Google forms was used to create the link for the questionnaire (illustrated in Table 1) that was distributed to the targeted population electronically via IDA to ensure uniform and validated distribution across all groups of dentists, including general practitioners, specialists, and consultants, throughout the country. Before distributing the questionnaire, a pilot study was conducted which included 36 dentists (about 10% of the sample size). Then data were entered on spreadsheet and double-checked by two authors which was followed pre-launch analysis was performed to check the internal consistency of all questionnaire's components.

**Table 1:** The study questionnaire.

**Age**					
**Country**					
Sex	A-Male	B-Female			
Qualification	A-Graduate	B-Postgraduate			
Designation	A-General practitioner	B-Specialist	C-Consultant		
Working place	A-Clinic	B-Hospital	C-Both		
Working type	A-Private	B-Government	C-Both		
1- Do you have a anxiety of being infected with COVID-19 by a patient or co-worker?				Yes	No
2- Are you afraid of providing treatment for any patient?				Yes	No
3- If a patient is coughing or suspected to be infected with COVID-19, are you afraid to provide treatment for him/her?				Yes	No
4- Do you anxious talking to the patients in close proximity?				Yes	No
5- Are you afraid that you could carry the infection from your practice back to your family?				Yes	No
6- Do you feel anxious when you hear that one of your co-workers or colleagues has been infected with COVID-19?				Yes	No
7- Do you know the illness problems associated with COVID-19 virus?				Yes	No
8- Do you know the mode of transmission of COVID-19 virus?				Yes	No
9- Are you updated with the current WHO guidelines for cross-infection control for COVID-19 virus?				Yes	No
10- Are you currently asking every patient if he/she has recently been in contact with an infected COVID-19 person?				Yes	No
11- Are you or your staff members taking every patient's body temperature before performing dental treatment?				Yes	No
12- Are you deferring dental treatment for patients with suspicious symptoms?				Yes	No
13- Do you think the routine surgical mask is effective to prevent COVID-19 cross infection?				Yes	No
14- Do you think that N-95 masks should be used routinely in dental practice because of the current COVID-19 outbreak?				Yes	No
15- Do you routinely follow universal infection control protocol for every patient?				Yes	No
16- Do you currently use rubber dam isolation for every patient as a part of your infection control?				Yes	No
17- Do you routinely use high volume section for every patient as part of droplets and airborne isolation precautions?				Yes	No
18- Do you routinely prepare antimicrobial mouth rinse for every patient to be used before starting treatment?				Yes	No
19- Have you changed or increased the procedure of infection control during the COVID-19 pandemic?				Yes	No
20- Has the schedule of your practice been changed to make it safer for you and the patient?				Yes	No
21- Do you routinely wash your hands with soap and water/ use sanitizer before and after treatment of every patient?				Yes	No
22- Do you and your staff members get tested for COVID-19 as a precautionary measure?				Yes	No
23- Do you know which authority to contact if you come across a patient with suspected COVID-19 infection?				Yes	No
25- What is the average drop in the number of patients visiting your practice as compared to the period before the COVID-19 pandemic?					
A- N/A	B- <25%	C- 25–50%	D- 50–75%	E- >75%	
26- How many appointments for non-urgent cases have you canceled recently as a part of COVID-19 precaution protocol?					
A- N/A	B- <25%	C- 25–50%	D- 50–75%	E- >75%	
27- Because of the COVID-19 pandemic, how much have the prices for your dental services been reduced, if at all?					
A- N/A	B- <25%	C- 25–50%	D- 50–75%	E- >75%	
28- If any, how much financial compensation (governmental and non-governmental) are you receiving for your losses in your practice?					
A- N/A	B- <25%	C- 25–50%	D- 50–75%	E- >75%	
29- To what extent have you reduced the staff numbers in your clinic?					
A- N/A	B- <25%	C- 25–50%	D- 50–75%	E- >75%	
30- By how much has the practice's income been reduced due to the COVID 19 pandemic?					
A- N/A	B- <25%	C- 25–50%	D- 50–75%	E- >75%	
31- What percentage of your stored dental materials have expired during the COVID-19 pandemic?					
A- N/A	B- <25%	C- 25–50%	D- 50–75%	E- >75%	
32- If applicable, what has been the average reduction of working days during the COVID-19 outbreak?					
A- N/A	B- <25%	C- 25–50%	D- 50–75%	E- >75%	

The questionnaire was adapted and modified from previously published surveys (19, 20). The questionnaire used for this study was composed of demographic/practice-related, closed end, and Likert five-point scale questions. These questions were divided into four sections:

Section 1 was designed to collect demographic/practice-related variables of the respondents.
Section 2, questions #1 to #6, was intended to assess the anxiety among dentists deriving from the COVID-19 infection.
Section 3, questions #7 to #23, was designed to evaluate the dentists' awareness and practice modification about the precautions and infection-control measures for COVID-19 infection.
Section 4, questions #24 to #32, consisted of questions that explored the economic impact of COVID-19 on dental practice.

For closed end questions, each positive response “Yes” was marked as “1” while “No” was marked with “0.” The frequency of the positive/negative responses was used to assess the dentists' anxiety (section 2) and awareness (section 3) regarding the COVID-19 infection. For section 4, the responses “N/A,” “ <25%,” “25–50%,” “50–75%,” “>75%” received sequential scores of “1,” “2,” “3,” “4,” “5,” respectively. The scores for each section were summed together to calculate the mean of the answers to evaluate the response according to the different independent variables.

### Statistical Analysis

Demographic data and total responses for each question were analyzed by descriptive statistics expressed by mean, standard deviation, and frequency/percentage. Inferential analysis for sections 2, 3, and 4 was performed by using Mann–Whitney test for comparing two groups while Kruskal–Wallis followed by *post-hoc* test was used for multiple groups comparisons. The statistically significant value was set at *p* < 0.05. All analyses were performed by using GraphPad Prism (Version 8.4.3, GraphPad Software, San Diego, CA, USA).

## Results

A total of 435 dentists (218 male and 217 female) with mean age of 36.51 ± 9.164 years (ranging from 23 to 70 years) participated in the study (Table 2). The number of respondents represented 89.9% of the calculated sample size (484) after excluding 49 dentists who did not response to the questionnaire within the specified time. The number of the respondents (435) was considered as a satisfactory response rate (89.8%). The majority of respondents (327, 75.2%) were graduate dentists, in comparison to 108 (24.8%) who had postgraduate degrees. The proportions of general practitioners, specialists and consultants were 47.8% (208), 47.4% (206), and 4.8% (20), respectively. Furthermore, 202 (46%) of respondents working in clinics worked in both the private and governmental sector (Table 2).

**Table 2:** Demographic characteristics of the study population.

**AGE (YEARS)**	
(mean± SD)	36.51 ± 9.16
Age range	23–70
**AGE GROUPS (YEARS)**	
≤35	222 (51)^§^
>35	213 (49)^§^
Gender	
Male	218 (50.1) ^§^
Female	217 (49.9)^§^
**QUALIFICATION**	
Graduate	327 (75.2)^§^
Postgraduate	108 (24.8)^§^
**DESIGNATION**	
General practitioner	208 (47.8)^§^
Specialist	206 (47.4)^§^
Consultant	21 (4.8)^§^
**WORKPLACE**	
Clinic	202 (46.4)^§^
Hospital	70 (16.1)^§^
Both	163 (37.5)^§^
**EMPLOYMENT TYPE**	
Private	135 (31.1)^§^
Governmental	98 (22.5)^§^
Both	202 (46.4)^§^
Total	435 (100)^§^

§ *Frequency, percentage*.

**Figure 1:**
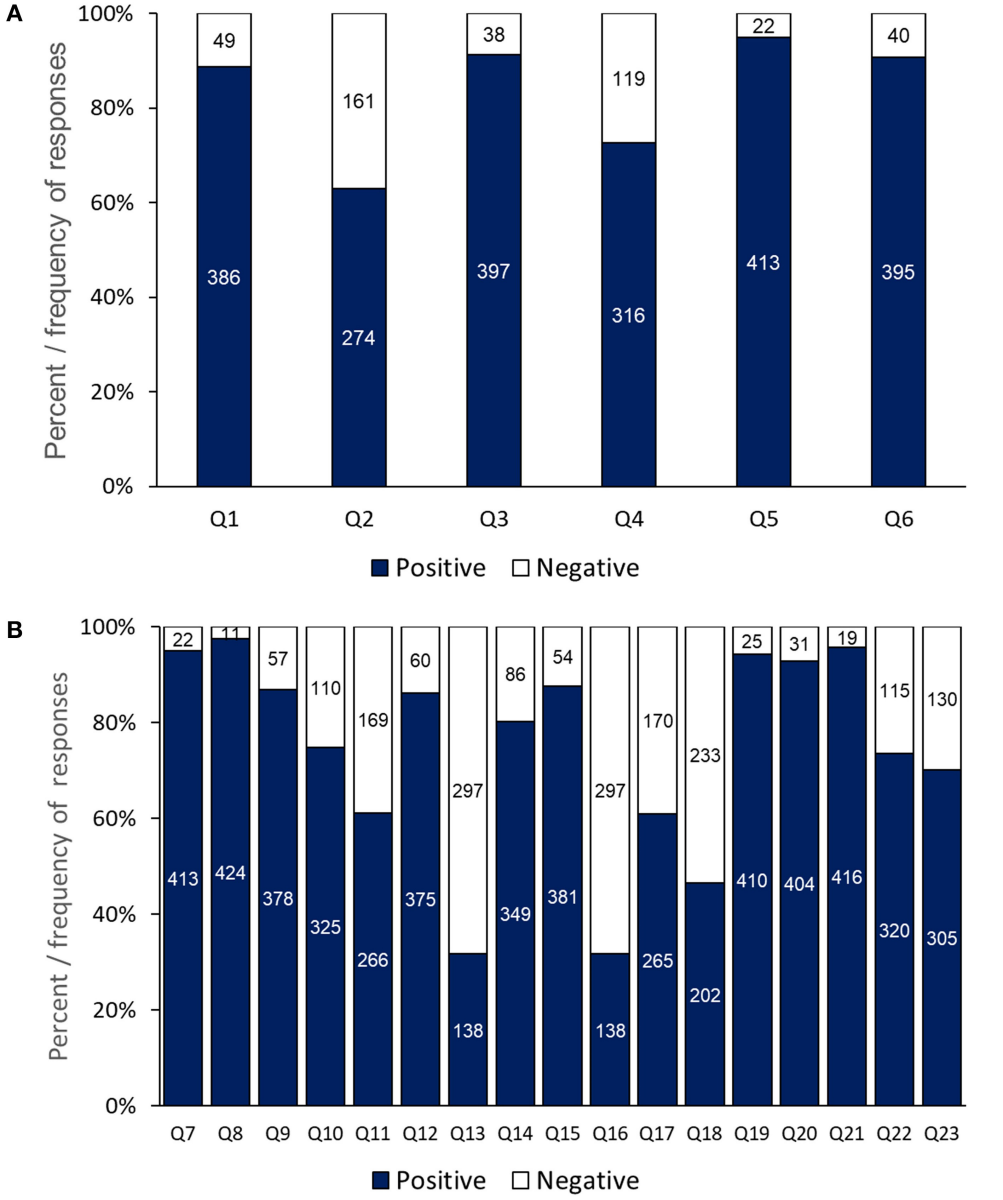
Dentists' responses to anxiety and awareness questions: **(A)** responses to individual questions on anxiety of COVID-19 and **(B)** awareness of precaution and infection control-measures.

Responses to section 2 questions relating to dentists' feelings about the COVID-19 pandemic indicated that the majority of respondents (386, > 80%) (Figure 1A) were anxious of catching the COVID-19 infection (Q1). Over 60% (274) of the dentists were afraid of treating any patients (Q2). This anxiety was further aggravated (397, 91%) if a patient was showing a sign of suspected infection such as coughing (Q3). Moreover, about 72% (316) of the respondents were not comfortable with being in close contact with their patients (Q4). The highest scoring response among the participants (413, 94%) was associated with the anxiety of carrying infection home to their family (Q5), whilst the second highest response (395, 90%) related to hearing that a co-worker had been infected with COVID-19 (Q6).

Regarding responses to section 3 questions, the dentists' highest scores related to their knowledge about the COVID-19 illness (Q7, 413, 94%) (Figure 1B) and its modes of transmission (Q8, 424, 97%), modification in infection control procedure (Q19, 410, 94%) re-scheduling patients' appointments (Q20, 404, 93%), and washing hands before and after treatment (Q21, 416, 95%). Additionally, three questions achieved (>80%) positive responses including: the dentist had updated information about the current WHO guidelines for infection control (Q9), deferring treatment of patients with suspicious symptoms (12), and following universal infection control protocol (Q15). Whereas, the lowest awareness score (138, 31.7%) related to the effectiveness of surgical masks to prevent cross infection (Q13), in comparison to nearly 80% of respondents who thought that N95 masks should be used routinely in dental practice (Q14). The response to (Q16) about the use of rubber dam as an infection control measure was equally low at 31.7% (138), but a higher percentage of respondents (265, >60%) confirmed that they used high volume section as a droplets precaution measure (Q17).

**Figure 2:**
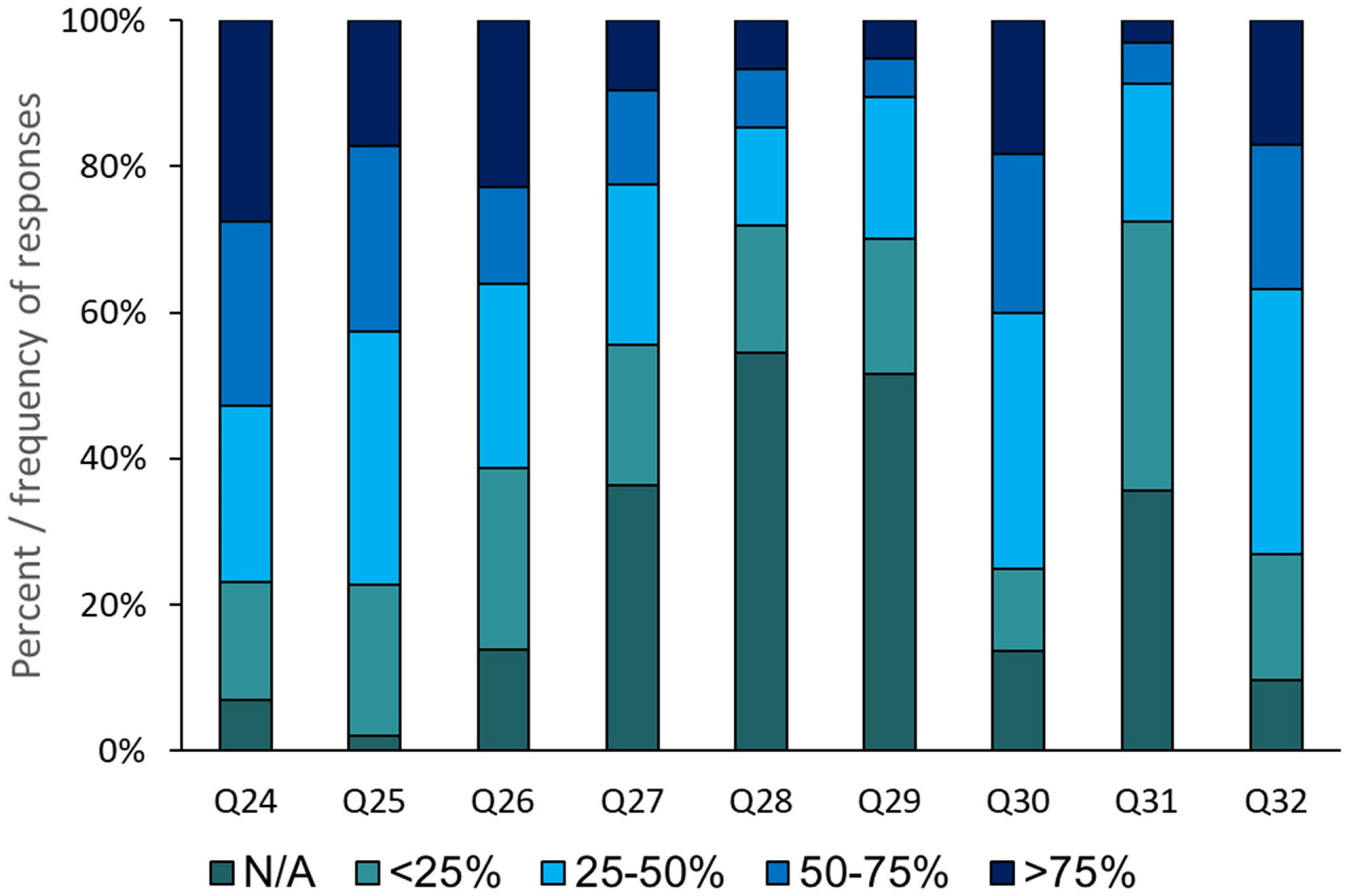
Dentists' responses on the economic impact of COVID-19 outbreak on their practice.

The economic impact of COVID-19 was investigated in this study via the section 4 questions (Figure 2). About 27% of the respondents suggested that the price of personal protection equipment had increased by >75% of the original price (Q24). Meanwhile, 32% of the dentists indicated that the number of patients had declined by 25–50% (Q25). The influence of COVID-19 on income was very apparent as >75% of the dentists reported that their income had dropped by 25–50% (Q30), with a similar response regarding the reduction in their working days (Q32). The level of financial compensation received by the dentists was unsatisfactory as more than half of the respondents were not eligible for any support programmes (Q28). However, the majority of the respondent dentists had not decreased their staff numbers (Q29) (Figure 2).

Inferential analysis of the questionnaire sections showed that older (>35 years old) and male respondents exhibited a statistically significant lower degree of anxiety of COVID-19 compared to younger (≤35 years old, *p* = 0.018) and female (*p* = 0.003) respondents, respectively (Table 3). Furthermore, the respondents who worked only in a hospital or in a clinic and hospital showed a statistically significantly higher anxiety than those working only in a clinic. Similarly, those working only in the government sector or in the government and private sector showed statistically significantly higher anxiety from COVID-19 than those working only in private clinics. However, respondents' qualifications and designation did not show a statistically significant impact on anxiety of COVID-19 (Table 3). Generally, the mean of responses showed a high level of anxiety of COVID-19 infection (5.01 ± 1.37; Table 3).

**Table 3:** Respondents' anxiety of the COVID-19 infection.

**Variables**	**Mean ± SD**	**Comparison**	***p*-value**
**AGE**			
≤35	5.14 ± 1.27	≤35 vs. >35	0.018^*^
>35	4.87 ± 1.45		
**GENDER**			
Male	4.78 ± 1.57	Male vs. female	0.003^*^
Female	5.24 ± 1.08		
**QUALIFICATION**			
Graduate	4.9 ± 1.39	Postgraduate vs. Graduate	0.363^*^
Postgraduate	5.04 ± 1.36		
**DESIGNATION**			
General practitioner (GP)	4.89 ± 1.43	GP vs. Specialist	0.377^†^
Specialist	5.11 ± 1.34	GP vs. Consultant	>0.999^†^
Consultant	5.23 ± 0.88	Consultant vs. Specialist	>0.999^†^
**WORKPLACE**			
Clinic	4.76 ± 1.45	Clinic vs. Both	0.003^†^
Hospital	5.31 ± 1.08	Clinic vs. Hospital	0.015^†^
Both	5.19 ± 1.33	Hospital vs. Both	>0.999^†^
**EMPLOYMENT TYPE**			
Private	4.71 ± 1.51	Private vs. Governmental	0.046^†^
Governmental	5.12 ± 1.09	Private vs. Both	0.015^†^
Both	5.2 ± 1.36	Governmental vs. Both	0.064^†^
Total	5.01 ± 1.37		

* *Mann–Whitney test*,

† *Kruskal–Wallis test*.

The mean awareness of respondents (section 3) was 12.65 ± 2.36 (Table 4). Age of the study participants have shown a statistically significant impact on respondents' awareness, whereas, this is not the case when male and female compared. Furthermore, qualification and designation (except for GP vs. Specialist) were found to have a statistically significant effect on respondents' awareness (*P* < 0.05, Table 4). Meanwhile, no statistically significant differences in respondents' awareness were identified according to workplace and employment type (Table 4).

**Table 4:** Respondents' awareness of COVID-19 infection-control measures.

**Variables**	**Mean ± SD**	**Comparison**	**p-value**
**AGE**			
≤35	11.14 ± 2.41	≤35 vs. >35	<0.001^*^
>35	12.99 ± 2.24		
**GENDER**			
Male	12.32 ± 2.58	Male vs. female	0.121^*^
Female	12.8 ± 2.09		
**QUALIFICATION**			
Graduate	11.98 ± 2.49	Postgraduate vs. Graduate	0.003^*^
Postgraduate	12.75 ± 2.28		
**DESIGNATION**			
General practitioner (GP)	12.48 ± 2.24	GP vs. Specialist	>0.999^†^
Specialist	12.53 ± 2.48	GP vs. Consultant	0.016^†^
Consultant	13.91 ± 1.92	Consultant vs. Specialist	0.027^†^
**WORKPLACE**			
Clinic	12.76 ± 2.07	Clinic vs. Both	0.569^†^
Hospital	12.44 ± 2.58	Clinic vs. Hospital	0.963^†^
Both	12.36 ± 2.58	Hospital vs. Both	>0.999^†^
**EMPLOYMENT TYPE**			
Private	12.82 ± 2.09	Private vs. Governmental	0.196^†^
Governmental	12.26 ± 2.38	Private vs. Both	>0.999^†^
Both	12.53 ± 2.52	Governmental vs. Both	0.674^†^
Total	12.65 ± 2.36		

* *Mann–Whitney test*,

† *Kruskal–Wallis test*.

Regarding the economic impact, no demographic variables emerged as having a statistically significant economic effect (Table 5). However, the mean economic effect was recorded as equal to 2.72 ± 0.71 out of 5 (Table 5), i.e., the economic losses incurred by the dental community amounted to more than 50%.

**Table 5:** Economic impact of the COVID-19 outbreak.

**Variables**	**Mean ± SD**	**Comparison**	***p*-value**
**AGE**			
≤35	2.71 ± 0.69	≤ 35 vs. > 35	0.955^*^
>35	2.72 ± 0.72		
**GENDER**			
Male	2.67 ± 0.72	Male vs. female	0.109^*^
Female	2.77 ± 0.69		
**QUALIFICATION**			
Graduate	2.66 ± 0.8	Postgraduate vs. Graduate	0.468^*^
Postgraduate	2.73 ± 0.67		
**DESIGNATION**			
General practitioner (GP)	2.71 ± 0.73	GP vs. Specialist	0.463^†^
Specialist	2.74 ± 0.65	GP vs. Consultant	0.463^†^
Consultant	2.54 ± 0.94	Consultant vs. Specialist	0.353^†^
**WORKPLACE**			
Clinic	2.73 ± 0.73	Clinic vs. Both	>0.999^†^
Hospital	2.68 ± 0.67	Clinic vs. Hospital	>0.999^†^
Both	2.71 ± 0.69	Hospital vs. Both	>0.999^†^
**EMPLOYMENT TYPE**			
Private	2.63 ± 0.74	Private vs. Governmental	>0.999^†^
Governmental	2.69 ± 0.67	Private vs. Both	0.077^†^
Both	2.79 ± 0.69	Governmental vs. Both	0.877^†^
Total	2.72 ± 0.71		

* *Mann–Whitney test*,

† *Kruskal–Wallis test*.

## Discussion

The present cross-sectional study reported a high level of anxiety among Iraqi dentists as a result of the COVID-19 outbreak and high awareness about preventing its transmission and avoiding infection; in addition, they and their practices have been economically affected due to this pandemic situation. These findings are understandable because dentists fall within the highest risk category, since their practice is associated with generation of droplets and aerosols which is considered as a main route of virus transmission (6). The high levels of anxiety recorded among these Iraqi dentists can be considered as natural human feelings during the pandemic situation, especially in light of the increasing infection and mortality rates. In Iraq the mortality rate is considered to be higher (about 3.9%) in comparison to other regional countries, such as the UAE with only a (0.5%) mortality rate (3). This could possibly be due to differences in available health resources between these two countries (21). The general weakness in the medical foundations and care system in Iraq after four decades of military conflicts (22), and the exaggerated pressure on the health care system due to the quick spread of the virus, general feelings of stress and fear among healthcare workers for their own safety and that of their families (23). Additionally, the nature of this disease, with its prolonged incubation period (as long as 14 days), its spectrum that ranged from asymptomatic to death, and the absence of a vaccine or treatment, are all factors potentially exacerbating stressful feelings among healthcare workers, especially dentists. This confirms findings from studies about COVID-19 (11), or previous outbreaks of similar infectious respiratory diseases such as SARS, which demonstrated severe and sustained psychological trauma, especially among the front line healthcare workers (24, 25).

Another interesting finding within the present study was that the recorded anxiety level was higher among younger than older dentists and females than males. This goes against the reports that among infected individuals there are higher risk groups, including older and male adults, who are more likely to develop severe respiratory symptoms and die than younger individuals and females (26). It may be that older dentists are more experienced than younger dentists in dealing with similar pandemic situations. This may make them more confident and less prone to anxiety. This was also reflected in the finding by this study of a statistically significant higher level of awareness about the virus and its mode of transmission among dentists aged above 35 years in comparison to those aged below 35 years. Moreover, since females, as mothers, tend to have closer contact with their children than other family members, the anxiety of transferring infection to family members, especially their children, could increase feelings of stress among female in comparison to male dentists. In the same way, dentists who were working in the public sector, in clinics or hospitals, reported higher anxiety levels than others. This could be attributed to the large number of dental patients visiting the public centers per day in comparison to private clinics (27). Consequently, this may increase dentists' concerns and anxiety of being infected compared to those working only in private clinics, who have more control over their appointments, case selection and cancellation of non-emergency cases. Finally, consultant dentists and those with postgraduate degree have higher levels awareness than their counterparts and this can be explained by the fact the majority of these dentists work in academic field, thus more update about the new developments in their field including the COVID-19 outbreak.

Almost all of the responding dentists were aware of the nature of the COVID-19 illness and its mode of transmission. This information is considered to be crucial in terms of applying infection control measures while carrying out dental treatment. In the same way, over 80% of the dentists responded that they were up to date about the current WHO guidelines for cross-infection control measures within dental practice (14). This was reflected through over 70% of the respondents asking their patients if they had been in contact with an infected COVID-19 individual, and above 60% recording patients' body temperature. Logically, this is basic information required during the routine examination to identify potentially infectious conditions among patients and the necessary precautionary management. Indeed, under such pandemic circumstances, the conventional precautions already recommended by WHO and any other infectious control authority worldwide should be rigorously applied to prevent cross-infection within dental practice. But unfortunately, more than 60% of the respondents were not using rubber dam for every patient as a cross-infection control. Rubber dam effectively limits the spread of aerosols during use of rotary instruments, decreases the hazard of fine instrument swallowing, gives excellent isolation of the working field and increases patients' acceptance of dental procedures (28). Therefore, training courses and workshops are suggested for Iraqi dentists, especially new graduates, to increase their awareness about the effectiveness of rubber dam in controlling the spread of infection. This could improve their hand skills and increase their willingness to consider rubber dam as part of their routine practice. The usage of high volume suction should also be considered as an essential method during routine dental practice to control aerosols and droplets evacuation (10).

The use of antimicrobial mouth washes before starting dental procedure is also recommended in the WHO guidelines for the current pandemic. Interestingly, over 40% of the respondents were complying with this recommendation. Mouthwashes such as chlorhexidine (29), 1% hydrogen peroxide (6), or povidone iodine (30) can be employed to decrease microbial loading inside the oral cavity. The latter has virucidal activity against SARS-CoV and MERS-CoV coronaviruses and is recommended to be used at 0.5% concentration as a mouthwash for patients before initiating a clinical procedure. Additionally, the operator is also advised to use povidone iodine as a nasal spray (0.4%) and mouthwash (0.5%) before and after suspected patient contact (31).

A positive finding by the current study was that the majority of Iraqi dentists were routinely focusing on hand hygiene before and after treating each patient, which is considered as an essential infection control measure for dental practitioners. Frequent hand washing with water and soap or using alcohol containing sanitizer is included in the WHO infection control guidelines for the current pandemic (14). The spread of respiratory viruses can be effectively avoided by proper hand washing and cleaning with alcohol-based sanitizers (8, 32). Furthermore, the majority of the study respondents agreed with the routine use of N-95 respirators rather than surgical masks in dental practice during the COVID-19 outbreak. The use of such personal protection equipment (PPE) is also recommended by the WHO and ADA guidelines when performing aerosols generating procedures (33).

Carefulness in selecting cases, controlling appointments, and receiving only emergency cases were also recommended by the ADA and WHO guidelines (13, 14). Almost all of the respondents in the current study stated that they had altered their appointment schedules to control the spread of the virus. This process could be started by initially calling patients or having video conferences to identify their need and decide if their condition requires clinical intervention (34). This could help in limiting face-to-face contact, making diagnoses through remote dental screening, deterring any COVID-19 susceptible patients, delaying nonemergency work, and planning effectively for the emergency cases (8, 34).

According to the findings of the present study, the economic losses caused by the COVID-19 outbreak to the dental community in Iraq amounted to about 50%. This is understandable during such a pandemic situation. The whole country has been affected by quarantines and lockdowns in an attempt to control the spread of the infection. The lockdowns consisted of intermittent periods of complete closure for all sectors followed by partial lockdown for specific sectors including schools, universities, tourism, and others. This has had severe economic impact on almost all activities, including dentistry (4). Additionally, the majority of respondents in the current study reported reduction of their working days, rescheduling of their appointments to see emergency cases only, absence of governmental support, and reduction in their total income. On the other hand, as some of the study participants received financial compensation, the degree of economic impact has shown to be varied from one to another dentist. However, none of demographical variables have shown an impact on income. This can be explained by the fact that during the national lockdown, working in private dental clinics was stopped by government and the only source of income were their monthly salary by the government. According to a recent investigation conducted in the U.S., this economic impact on dental services could be extended to 2022 because of financial hardship among dental patients (35). These financial impacts on dentistry as a profession may have serious implications for the future of this career.

The limitations of this study that should be considered is the rapid changes in respondents' psychology and practice in accordance with the progression of the current outbreak, the attitudes and awareness of dentists will certainly be altered by future alteration in the scientific knowledge about COVID-19. Additionally, although the distribution of the questionnaire for the present study was done through the IDA, fewer responses were obtained from consultants in comparison to the other designations. This possibly because of the general panic situation during the COVID-19 outbreak altered the priorities for potential respondents. Thus, the findings of the current study should be carefully interpreted to avoid generalization of the data.

## Conclusions

The emergence of the novel coronavirus has increased concern among healthcare workers, especially dentists, regarding aerosols borne microbes rather than the conventional blood borne microbes. This has dramatically increased the anxiety among dentists about getting the infection and has altered their awareness toward a new era. Although, Iraqi dentists have gained a high level of knowledge and practice to address the COVID-19 outbreak, their anxiety was high. It is important in the current scenario to modify the conventional dental practice to deal with emergencies only or close down practices until the outbreak recedes. However, this situation may last for an indefinite period, which would have a dramatic impact not only on the economy which have shown to affect the majority of responders by reducing their income by 50% but also on the future of the dental profession such as increasing levels of anxiety amongst dentist and adapting to practice modification.

## Data Availability Statement

The raw data supporting the conclusions of this article will be made available by the authors upon reasonable request.

## Ethics Statement

The study was approved by the ethics committee of the College of Dentistry, University of Baghdad in compliance with the Helsinki declaration.

## Author Contributions

AM: study conception. AA and SG: study design. AM, AA, SG, and SQ: data collection. AA and SG: data analysis and manuscript drafting. AA and SG: data interpretation. SG and SQ: critical revision of the manuscript. All authors: approval of the final version. All authors contributed to the article and approved the submitted version.

## Conflict of Interest

The authors declare that the research was conducted in the absence of any commercial or financial relationships that could be construed as a potential conflict of interest. The reviewer FL declared a shared affiliation with several of the authors AA and AM to the handling editor at time of review.
